# Population Growth Potential of the Bed Bug, *Cimex lectularius* L.: A Life Table Analysis

**DOI:** 10.3390/insects2020173

**Published:** 2011-04-29

**Authors:** Andrea M. Polanco, Carlyle C. Brewster, Dini M. Miller

**Affiliations:** Department of Entomology, Virginia Tech, 216A Price Hall, Blacksburg, VA 24061, USA; E-Mails: apolanco@vt.edu(A.M.P.); dinim@vt.edu(D.M.M.)

**Keywords:** *Cimex lectularius*, bed bug, life table, life history, resistance, survivorship, development, pyrethroids

## Abstract

Experimental life tables were constructed and analyzed for three strains of the common bed bug: a pyrethroid-susceptible laboratory strain (HS), a highly resistant field strain (RR), and a field strain with a declining level of resistance (KR). Egg to adult survival in the RR strain was 94% compared with 79% and 69% in the HS and KR strains, respectively. The RR strain also developed significantly faster from egg to adult (∼35 days) than the other two strains (∼40 days). Analysis of a survivorship and fecundity life table for the RR strain produced the following results. The average life expectancy for a newly laid egg was ∼143 days, and that of a newly molted adult was ∼127 days. Females produced an average of 0.64 daughter eggs/day with the highest weekly production during the fifth week of adult life. Analysis of daily reproductive parity showed that females produced 1–3 and 4–6 eggs on 79 and 21% of the days, respectively, when egg laying occurred. The net reproductive rate (*R_0_*) of the RR strain was ∼35, which represents a 35-fold increase in the population per generation (∼92 days). The intrinsic rate of increase, *r*, was 0.054 indicating that the population multiplies 1.1 times/female/day (λ) and doubles in size every 13 days. The stable age distribution (*c_x_*) was dominated by nymphs (54%), followed by eggs (34%) and adults (12%). Reproductive values (*v_x_*) for the strain increased from egg to the adult stage.

## 1. Introduction

During the 1940s, a new synthetic organochloride insecticide, dichlorodiphenyl trichloroethane (DDT), was developed primarily to control insect vectors of diseases such as malaria and typhus in military and civilian populations [1]. Because of its long residual activity and low cost, DDT was also used to control infestations of other insect pests of humans, including the bed bug, *Cimex lectularius* [2]. The widespread use of DDT in the 20th century essentially eradicated bed bug populations from the U.S. [1,3–5]. However, at the start of the 1990s, there were growing concerns about bed bugs in the United States with pest management companies experiencing an exponential increase in complaints of infestations [1].

The majority of insecticides available for bed bug control in the 1950s and 1960s, including DDT, are no longer in use today. Recent cancellation by the Environmental Protection Agency (EPA) of many organophosphate and carbamate insecticides labeled for indoor use has also narrowed the list of insecticidal products for the bed bug [6]. Currently, the primary insecticides for bed bug control belong to the pyrethroid class, but repeated applications of these chemicals over time have led to the development of resistance in the insect [6–9]. Moore and Miller [7], for example, studied a field strain of bed bugs resistant to pyrethroids and found that individuals could survive long periods of exposure (>343 h) to dried residues on treated panels.

Because the use of chemicals is the primary strategy for managing modern bed bug infestations, it is important to understand the mechanisms of resistance in the insect [9]. As equally important for management is an understanding of the biology and ecology of the bed bug [10] and specifically, differences between pyrethroid-susceptible and resistant strains. Indeed, some of the biological characteristics of *C. lectularius* were described previously [2,11], as were aspects of the ecology of the insect [12,13]. However, while the biological and ecological information provided by previous studies is still valuable, it may be unwise to assume, from a management perspective, that the bed bug populations currently infesting the United States are exactly the same as those that existed >70 years ago.

One of the tools available to entomologists for gathering basic information on the biology, ecology, and population dynamics of an insect is the life table [14]. Both experimental (for studying populations reared under controlled laboratory conditions) and ecological (for populations in their natural environment) life tables measure biological attributes, such as survivorship (or mortality) and fecundity within a population [15,16]. Life tables are also used to determine the age structure of the population, reproductive contribution of the different life stages to future population size, and the maximal rate of increase at any particular combination of environmental factors [15,16]. It is not surprising, then, that in the entomological literature, one can find studies of the development and application of life tables for species in many orders including Lepidoptera [17,18], Diptera [19,20], and Hymenoptera [21]. Life tables have also been used to study the life history of mite species [22]. To our knowledge, no life tables have been developed for the common bed bug, and there is only one study [23] in which a life table was developed for a parasitic insect of humans.

The purpose of this study was to construct experimental life tables to document and quantify the life history characteristics of three bed bug strains representing different levels of resistance to pyrethroid insecticides. One bed bug strain was highly susceptible to pyrethroid insecticides, the second was a highly resistant strain, and the third showed a declining level of resistance to pyrethroids. Cohort life tables were developed to study the survivorship and development of the immature stages of all three bed bug strains. In addition, a complete survivorship and fecundity life table was developed for the pyrethroid-resistant strain.

## 2. Experimental Section

### 2.1. Bed Bug Strains and Rearing

This study examined the life history characteristics of three bed bug strains. The first strain, Harlan, was a laboratory strain acquired from Dr. Harold Harlan (National Pest Management Association, Fairfax, VA) in February 2005. Dr. Harlan maintained this population of bed bugs for >37 years. Harlan (HS) represented our pyrethroid-susceptible strain (Table 1). The second bed bug strain was the field strain, Kramer, which was collected in 2006 from a multiple-unit apartment building in Arlington, VA. The third strain, Richmond, was also a field strain collected in September 2008 from a group home for adult residents in Richmond, VA. Both the Kramer (KR) and Richmond (RR) strains initially demonstrated high levels of resistance to pyrethroids compared with HS (Table 1). However, the KR strain appears to be losing resistance (Table 1), and as such, represents a strain with a declining level of resistance to pyrethroid insecticides.

Colonies of the three bed bug strains were maintained in the Dodson Urban Pest Management Laboratory at Virginia Tech, Blacksburg, VA. Individuals were reared in plastic jars covered at one end with a cloth mesh. Two pieces of cardboard were placed inside the jars to allow bed bugs to crawl onto and feed through the mesh. Feeding was carried out once a week using an artificial feeder containing chicken blood formulated with sodium citrate as an anti-coagulant and maintained at 35.5 °C by circulating hot water. Between feedings, the jars with bed bugs were stored in an environmental chamber at 26.1–26.5 °C, 68.9% RH, and photoperiod of 12:12 h, L:D. These conditions were within the range at which Johnson [13] evaluated *C. lectularius* populations for maximum fecundity and longevity. None of the colonies were exposed to insecticides while in the laboratory.

### 2.2. Bed Bug Survivorship and Development

Fifth instar (N5) males and females were selected from each of the three bed bug strains (HS, KR, and RR), and were fed and allowed to molt to the adult stage. Feeding was carried out by inverting the N5 nymphs in their respective jars and placing the mesh top against the arm of a human volunteer. This feeding protocol was approved by the Virginia Tech Institutional Review Board (IRB 06–165). Once N5 nymphs molted to adults, they were sorted into couples (male and female) and allowed to feed on human blood. Once fully engorged, the couples were placed in Petri dishes (Fisher brand 60 × 15 mm) containing a single filter paper (Whatman 42.5 mm) and left to produce eggs. Eggs produced on the same day by couples of each bed bug strain represented a cohort. Survival and development of eggs in each cohort were then observed and recorded daily through adulthood, with individuals fed on human blood approximately every 7 days as described above. A total of 175, 198, and 192 eggs were followed in constructing the survivorship (egg to adult) life tables for the HS, KR, and RR bed bug strains, respectively. All studies of the life history of the KR strain were conducted in 2009, three years after the strain was initially tested for pyrethroid-resistance; the RR strain was studied about one month after being tested for resistance in February 2009 (Table 1).

### 2.3. Survivorship and Fecundity in the Bed Bug Field Strain

Because of the logistical challenges of feeding and observing a large number of individuals from egg to the end of the adult stage, we elected to follow a smaller cohort of 49 eggs of the RR strain to study the survivorship and fecundity characteristics of a field strain. Observations of survival and development were made for each egg into adulthood using the rearing and feeding protocols described above. Once individuals entered the adult stage, males and females were placed in individual pairs in Petri dishes (Fisher brand 60 × 15 mm) containing filter paper (Whatman 42.5 mm). The couples were fed on human blood and left to produce eggs. The number of eggs produced was recorded daily until egg laying ceased, after which adults were given another blood meal. In general, adults were fed approximately every 10 days, and egg laying was quantified until all adult female bed bugs died.

### 2.4. Life Tables and Data Analysis

Data on survival and development for the immature stages (egg to adult) of the three bed bug strains (HS, KR, and RR) were used to construct survivorship life tables, while data from the evaluation of the smaller cohort of the RR strain were used to develop a complete (survivorship and fecundity) life table. All of the parameters and formulae for constructing and analyzing the life tables can be found in Carey [26], Leslie and Park [27], and Birch [28]. The mean (± 95% CI) duration of development for each of the five immature stages was calculated using the methods described in Carey [26] and Pontius *et al.* [29]. Significant differences in the development duration for each life stage among the three bed bug strains were determined by the non-overlapping confidence intervals.

The data in the complete life table for the smaller cohort of the RR strain were used to estimate the intrinsic rate of increase of the population, *r*, using both the approximate and more precise iterative methods [28]. Also derived were the population stable stage distribution (*c_x_*), stage-specific reproductive value (*v_x_*), gross reproductive rate (*GRR*), net reproductive rate (*R_0_*), mean generation time (*T*), and population doubling time (*DT*). In addition, we examined the daily reproductive parity for the strain to measure of the consistency (or heterogeneity) in egg-laying among females [26].

Several assumptions were made in developing the life tables. We assumed that all of the eggs laid were fertile and that failure to hatch was due to natural mortality and not accidental damage during handling. Because the sex of individual bed bugs could not be determined in the egg or immature stages, we also assumed that mortality rates in these stages were equally applicable to males and females. Finally, we assumed a 1:1 sex ratio of male and females, an assumption which is in keeping with previous observations of laboratory-reared bed bugs [13].

## 3. Results

### 3.1. Bed Bug Survivorship (l_x_)

The survivorship (egg to adult stage) life tables for the Harlan (HS), Kramer (KR), and Richmond (RR) bed bug strains are combined and presented in abridged form in Table 2. For conciseness, and because only one column of data is required to calculate all other information in a life table, only selected columns in a typical life table are shown along with stage-specific mortality. Several life history characteristics of the three bed bug strains are evident in the life table. The RR strain had the highest immature survivorship. Egg to adult survival (*l*_Adult_*/l_0_*) for the HS, KR, and RR strains were ∼79%, 69%, and 94%, respectively. Stage-specific mortality was relatively high for the egg and N1 stages of the HS and KR strains compared with the RR strain. However, mortality within the N2 to N5 stages was relatively low for all three strains (Table 2).

Another interesting life history characteristic can be seen by examining the *e_x_* column of each life table. Life expectancy for a newly produced egg of the HS, KR, and RR strain to the adult stage was 35.3, 32.2, and 34.4 days, respectively. This implies that, on average, a newly laid egg from the HS and RR strains is expected to be alive ∼2–3 days longer before becoming an adult compared with an egg of the KR strain. However, once an individual of the RR strain reaches the N5 stage, it is expected to have ∼2 more days of life remaining before entering the adult stage compared with an N5 individual of the HS and KR strains.

### 3.2. Bed Bug Development

Table 3 shows the mean (±95% CI) duration of development for the immature life stages of the three bed bug strains. No significant difference (*p* > 0.05) was observed between the overall development durations for the HS and KR strains. However, the development durations for the two strains differed significantly (*p* < 0.05) from those of the RR strain. The development durations in the egg and N5 stages were significantly longer, and the developmental duration of the N1–N4 stages significantly shorter in the RR strain compared with the HS and KR strains. Overall, egg to adult development duration for the RR strain was shorter by ∼5 days than for the other two strains.

### 3.3. Survivorship and Fecundity Life Table (RR strain)

An abridged survivorship and fecundity life table for the RR strain is presented in Table 4. We found no significant differences between the stage-specific development durations for the smaller RR cohort and those of the larger cohort reported in Table 3. This suggests that the transition dynamics of the immature stages of the smaller cohort (Tables 4) were similar to those of the larger RR cohort (Table 2). Table 4 also shows that the average life expectancy (*e_x_*) of a newly laid egg was ∼143 days, with a newly molted adult female having a life expectancy of ∼127 days. Six females survived as adults for 191 days (227 total days of life), with all females tested having a range of life (egg to adult death) of 84–227 days.

**Figure 1:**
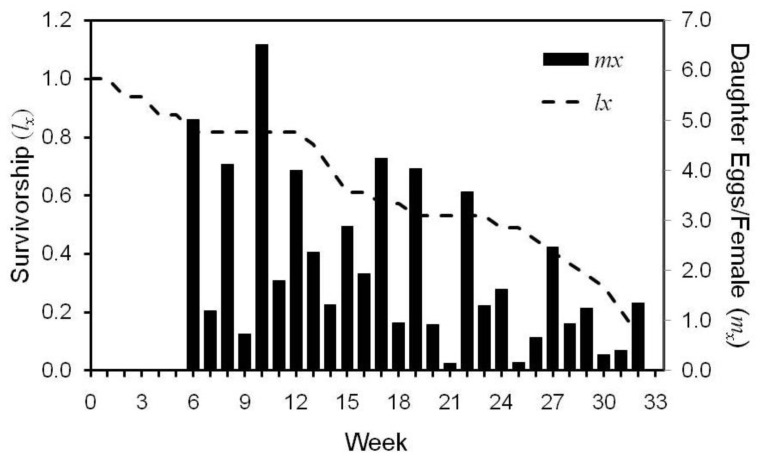
Survivorship curve (*l_x_*) and the number of daughter eggs produced weekly by each female (*m_x_*) of the pyrethroid-resistant Richmond (RR) bed bug strain.

The average daily output of eggs by a single female during her entire lifetime was 0.64 which roughly equates to a female producing one female egg every 2 days. The highest weekly egg production per female was observed during week 10 or the fifth week of adult life (Figure 1), with the highest *m_x_* value (2.08) observed at 78 days of age (42 days of adult life). Analysis of the daily reproductive parity showed that there were a total of 2541 female-days in the cohort of which 1829, 562, 147, and 3 days were spent laying 0, 1–3, 4–6, and 7 eggs, respectively. As such, for the entire adult period 0, 1–3, 4–6, and 7 eggs were laid on 72.0, 22.1, 5.8, and 0.1% of the female-days, respectively. When we considered only days in which at least one egg was laid, the distribution of egg production was 79.0% for 1–3 eggs, 20.6% for 4–6 eggs, and 0.4% for 7 eggs.

The *GRR* for the RR strain was ∼53 daughter eggs per female (Table 4). When female survivorship was considered, the RR strain had a net reproductive rate, *R_o_*, of ∼35 daughter eggs per female. This suggests that in a population of the RR strain the average female will be replaced by 35 daughters. That is, the RR strain is expected to have a 35-fold increase in the population per generation, with a mean generation time, *T*, of ∼92 days. The approximate value of the intrinsic rate of increase, *r*, for RR was 0.039 daughters/female/day. However, the precise estimate of *r* calculated using the iterative formula [28] was 0.054 daughters/female/day. This indicates that a population of the RR bed bug strain multiplies ∼1.1 times per female per day (*i.e.*, *λ*) and will double in size approximately every 13 days. In addition, if conditions remained constant, we should expect, based on the stable age distribution, *cx*, that the majority of individuals in the population will be in one of the five nymphal stages (∼54%), followed by the egg (∼34%), and adult (∼12%) stages. The analysis also showed that the relative reproductive contribution (*v_x_*) of individuals to future population size increases from egg to adult stage.

## 4. Discussion

Our study showed that on a regular feeding schedule, individuals of the highly resistant RR bed bug strain had a higher egg to adult survivorship than the susceptible HS strain and the moderately resistant KR strain. The RR strain also developed significantly faster from egg to adult compared with the other two strains. These results are interesting particularly in light of reports of reduced fitness (increased fitness costs) in insecticide-resistant populations of several insect species [30–33]. Individuals from resistant populations generally have been shown to have longer developmental times and reduced fecundity compared with susceptible individuals [30–33] partly because of the energy that resistant individuals must invest in insecticide detoxification [34]. However, as Georghiou and Taylor [34] pointed out, and others have noted [35], there are exceptions to the idea of resistance and fitness costs, with considerably variation among species and insecticides [36]. The common bed bug may be one of the exceptions, but this can only be confirmed with additional studies using strains from different environments.

The life tables, and the survivorship and development data obtained from these life tables, show that the HS and KR strains have similar life history characteristics suggesting that the resistance level of the KR strain is closer to that of the HS strain than to the RR strain. Table 1 shows that within five years, there was a significant decline in the resistant ratio of the KR strain to Suspend SC. This phenomenon of declining resistance is one that is commonly seen with insects taken from the field and reared for several generations in the laboratory [37,38]. The KR strain has been reared in the laboratory since 2006. Assuming that this strain had the same mean generation time as the RR strain (92 days), then the KR strain would have been in the laboratory for about 12 generations at the time the life table studies were conducted. Eleven generations is all that it would take for the German cockroach to lose resistance to a pyrethroid insecticide with no selection pressure [38].

The present investigation also provides baseline information on the expected longevity and fecundity of adults, and on the population structure and growth potential of a field strain of the bed bug. Females of the RR field strain had an average life expectancy (*e_x_*) of ∼127 days at 26 °C, 69% RH with regular feedings (every 10 days). This value is much lower than the lifespan of bed bug adults reported elsewhere. Adult bed bugs were reported to survive for 3–4 years with only occasional feedings [12], and when fed regularly, survived >326 days at 22 °C [39]. One explanation for the difference may be that the adult bed bugs studied in the 1930s and 1940s were not resistant to insecticides, a condition that has been shown to also affect adult longevity.

The positive intrinsic rate of increase (*r*) for the RR strain (0.054) indicates that under constant conditions the population will continue to grow. Positive *r*-values are not uncommon for insect and mite species [23,40] The *r*-values compiled by Taylor [40], for example, were positive for all species and ranged between 0.04 and 0.31. The two species listed by Taylor [40] in the order Hemiptera had *r*-values of 0.074 and 0.060. The *r*-value for the bed bug falls within this range and is comparable with that of the German cockroach, *Blattella germanica* (L.), which has an *r*-value of 0.06 [41]. German cockroaches are prolific reproducers and are very successful at infesting human environments. Based on the *r*-value for the RR strain we could also assume that populations of this strain would be equally successful at proliferating in homes and other residential dwellings.

A practical value of life tables is to provide information on the life history of the species and to aid in the development of control measures [14,17,42]. It is clear from the life tables developed here that the bed bug has a relatively low natural mortality and rapid population growth (*DT* = 13 days). We also found that under constant conditions the stable age distribution of a bed bug population will be dominated by eggs and nymphal stages. Although Taylor [40] cautioned that a stable age distribution (SAD) is unlikely in natural populations, the SAD still provides useful information for making management decisions, particularly with respect to sampling [15]. A high percentage of individuals in the egg and immature stages of the SAD has often been observed for insect and mite species [22,23,28]. However, depending on the population sampling method used, the numbers of adults obtained in a sample can lead to incorrect conclusions of the true structure and size of the population. Sampling methods for the bed bug are needed that can account for the abundance of eggs and early immature stages in the population, especially since survivorship within these stages is relatively high.

This study is a first step in characterizing the life history, growth potential, and structure of modern bed bug populations in the United States. However, in the future, it will be necessary to develop life tables to assess other field strains of the bed bug. By improving our knowledge of the biological characteristics of these populations we may be able to design prevention measures to inhibit the proliferation of the bed bug, and reduce their current rate of spread. For example, one of the uses of life tables described in early literature is as a source of information for the development of population models [14]. Accordingly, life tables can be used to identify and evaluate important mortality-causing factors, which are then incorporated into models for studying the behavior of the population under specific conditions [14]. We are already in the process of developing a model for the bed bug using the data presented in this study.

## 5. Conclusions

The only study we could find on the use of life tables as a source of basic information on the biology and growth potential of a parasitic organism of humans was that for the human louse, *Pediculus humanus* L. [23]. The present study provides life tables for another parasitic insect of humans, the common bed bug. Three stains of the bed bug were studied: a pyrethroid-susceptible laboratory strain (HS), a highly resistant field strain (RR), and a field strain with a declining level of resistance (KR). Higher survivorship and shorter development duration were observed in the highly resistant RR strain compared with the other two strains that had moderate to high levels of susceptibility to pyrethroid insecticides. Adult female life span was also shorter than has been reported in the literature. The study also quantified and showed that field populations of the bed bug have a high growth rate, which is comparable with another urban pest, the German cockroach. Like many other pest insects, the stable age distribution (SAD) of the bed bug is dominated by eggs and nymphs, a fact that has implications for sampling and estimation of population size.

## Figures and Tables

**Table 1:** Time to mortality of adults from three strains of the bed bug, *Cimex lectularius*, bioassayed for resistance to pyrethroid insecticides *.

**Bed Bug Strain [Date Collected]**	**Date Tested**	**Treatment**	**LT_50_** **(95%** **CL) (Hours)**	**Resistance Ratios**	**Reference**
Harlan (HS) [February 2005]	June 2006	Suspend SC	1.01 (1.00–1.15)	---	[7]
		Dragnet	1.46 (1.37–1.56)	---	
	February 2009	Suspend SC	0.82 (0.72–0.94)	---	[24]
		Dragnet	1.48 (1.23–1.78)	---	
	February 2011	Suspend SC	3.00 (0.90–7.10)	---	[25]
Kramer (KR) [January 2006]	June 2006	Suspend SC	343.48 (328.28–358.81)	339.6	[7]
		Dragnet	>168.00	>115.1	
	February 2011	Suspend SC	11.48 (7.53–18.25)	3.83	[25]
Richmond (RR) [September 2008]	February 2009	Suspend SC	320.24 (304.40–338.18)	390.5	[24]
		Dragnet	>432.00	>291.7	

* Notes: Harlan was collected from a population in the laboratory since 1973; Kramer and Richmond are field collected strains. Tests on both field strains (Kramer and Richmond) and the laboratory strain (Harlan) were run on the same day for comparison using a 1:1 male to female ratio. Active ingredient in Suspend SC is deltamethrin (0.06%); active ingredient in Dragnet is permethrin (0.05%).

**Table 2:** Abridged survivorship life tables for the immature stages of Harlan (HS), Kramer (KR), and Richmond (RR) strains of the common bed bug, *Cimex lectularius* *.

**Harlan (HS)**						**Kramer (KR)**						**Richmond (RR)**					
***x***	**Life Stage**	***n****_x_*	***l****_x_*	***e****_x_*	**Stage Mortality (%)**	***x***	**Life Stage**	***n****_x_*	***l****_x_*	***e****_x_*	**Stage Mortality (%)**	***x***	**Life Stage**	***n****_x_*	***l****_x_*	***e****_x_*	**Stage Mortality (%)**
0	Egg	175	1.000	35.3	9.7	0	Egg	198	1.000	32.2	12.1	0	Egg	192	1.000	34.4	1.0
6	N1	158	0.903	32.6	6.3	6	N1	174	0.879	29.9	15.0	6	Egg	190	0.990	28.7	
7	N1	154	0.880	32.4		7	N1	170	0.859	29.6		7	N1	190	0.990	27.7	1.6
11	N1	153	0.874	28.6		11	N1	161	0.813	27.1		11	N2	187	0.974	24.1	0.0
14	N2	148	0.846	26.6	2.7	14	N2	148	0.747	26.4	2.7	14	N2	187	0.974	21.1	
17	N2	146	0.834	23.9		17	N2	144	0.727	24.2		17	N3	187	0.974	18.1	1.6
22	N3	144	0.823	19.2	2.1	22	N3	144	0.727	19.2	1.4	22	N3	186	0.969	13.2	
23	N3	144	0.823	18.2		23	N3	144	0.727	18.2		23	N4	184	0.958	12.3	2.1
29	N3	141	0.806	12.5		29	N4	142	0.717	12.3	0.7	29	N5	180	0.938	6.5	0.0
30	N4	141	0.806	11.5	0.7	30	N4	142	0.717	11.3		30	N5	180	0.938	5.5	
35	N4	141	0.806	6.5		35	N4	141	0.712	6.4		35	Adult	180	0.938		
37	N4	141	0.806	4.4		37	N5	141	0.712	4.4	3.5	37					
38	N5	140	0.800	3.5	0.7	38	N5	140	0.707	3.4		38					
41	Adult	139	0.794			41	Adult	136	0.687			41					

* Notes: N1 = 1^st^ instar; N2 = 2^nd^ instar; N3 = 3^rd^ instar; N4 = 4^th^ instar; N5 = 5^th^ instar. *x* is age in days, *n_x_* is the number of individuals alive at age *x*, *l_x_* is the probability of being alive or surviving to age *x*, and *e_x_* is the expectation of life or life expectancy of an individual alive at age *x*.

**Table 3:** Mean (± 95% CI) duration of each life stage (days) for two pyrethroid-resistant (KR and RR) and one susceptible strain of the common bed bug, *Cimex lectularius.*

**Life Stage**	**Bed Bug Strain (*n*** **=** **Life Table Cohort Size)**				

	**Harlan (HS) (*n*** **=** **175)**	**Kramer (KR) (*n*** **=** **198)**	**Richmond (RR) (*n*** **=** **192)**
Egg	5.41 (± 0.08) a	5.84 (±0.11) b	6.33 (± 0.09) c
1^st^ instar (N1)	7.82 (± 0.21) a	7.95 (± 0.24) a	4.46 (±0.11) b
2^nd^ instar (N2)	8.20 (± 0.27) a	7.80 (±0.31) a	6.02 (±0.12) b
3^rd^ instar (N3)	8.02 (± 0.29) a	7.02 (± 0.34) b	5.62 (±0.17) c
4^th^ instar (N4)	7.63 (± 0.29) a	7.61 (±0.33) a	5.66 (±0.19) b
5^th^ instar (N5)	3.28 (± 0.22) a	4.12 (±0.24) b	6.73 (±0.19) c
Egg – Adult	40.37 (±0.19) a	40.35 (±0.19) a	34.82 (±0.12) b

* Notes: Means followed by the same letter in each row are not significantly different (*P* > 0.05).

**Table 4:** Abridged survivorship and fecundity life table, and associated life history parameters, for the pyrethroid-resistant Richmond (RR) field strain of the common bed bug, *Cimex lectularius* *.

**Age,** ***x*** **(Days)**	**Life Stage**	***l****_x_*	**e_x_**	***m****_x_*	***c****_x_*	***v****_x_*
0	Egg	1.000	142.5		33.5	1.00
7	N1	1.000	135.5		13.8	1.46
11	N2	0.939	140.3		15.3	1.81
17	N3	0.939	134.3		11.1	2.50
23	N4	0.939	128.3		8.9	3.46
30	N5	0.878	129.9		5.1	5.04
35	Adult	0.857	126.9	0.00	12.4	6.97
39	Adult	0.816	130.2	0.05		
89	Adult	0.776	84.7	0.61		
92	Adult	0.735	86.2	0.46		
93	Adult	0.694	90.3	0.60		
104	Adult	0.612	90.2	1.45		
115	Adult	0.571	84.9	0.07		
130	Adult	0.531	75.5	0.69		
165	Adult	0.490	44.2	0.33		
184	Adult	0.449	27.9	0.50		
185	Adult	0.408	29.7	0.62		
198	Adult	0.365	18.9	0.33		
199	Adult	0.327	20.3	0.29		
219	Adult	0.163	6.5	0.25		
220	Adult	0.122	7.5	0.43		
227	Adult	0.122	0.5	0.00		
228	Adult	0.000				
Gross Reproductive Rate					*GRR*	52.67
Net Reproductive Rate					*R_0_*	34.92
Mean generation time (days)					*T*	91.52
Intrinsic rate of increase (female/female/day)			ln *R*_0_/*T*		*r*	0.039
			Σ*e*^−^*^rx^l_x_m_x_*=1			0.054
Finite rate of increase (female/female/day)					*λ*	1.055
Doubling Time (days)					DT	12.86

* Notes: N1 = 1^st^ instar; N2 = 2^nd^ instar; N3 = 3^rd^ instar; N4 = 4^th^ instar; N5 = 5^th^ instar. *l_x_* is age-specific survivorship, *e_x_* is life expectancy for an individual alive at age *x*, *m_x_* is the mean number of daughter eggs per female alive at age *x*, *c_x_* is the stable age distribution, and *v_x_* is the reproductive value.

## References

[1] Potter M.F. (2008). Bed bug supplement. The history of bed bug management. Pest Control Technol..

[2] Usinger R.L. (1966). Monograph of Cimicidae: Hemiptera-Heteroptera.

[3] Harlan H.J. (2006). Bed Bugs-Importance, Biology and Control Strategies.

[4] Harlan H.J. (2007). Bed bug control: Challenging and still evolving. Int. Pest Control..

[5] Cooper R. (2006). Bed bugs-still more questions than answers: A need for research and public awareness. Am. Entomol..

[6] Potter M.F. (2006). The perfect storm: An extension view on bed bugs. Am. Entomol..

[7] Moore D.J., Miller D.M. (2006). Laboratory evaluations of insecticide product efficacy for control of *Cimex lectularius*. J. Econ. Entomol..

[8] Romero A., Potter M.F., Potter D., Haynes K.F. (2007). Insecticide resistance in the bed bug: A factor in the pest's sudden resurgence?. J. Med. Entomol..

[9] Yoon K.S., Kwon D.H., Strycharz J.P., Hollingsworth C.S., Lee S.H. (2008). Clark. J.M. Biochemical and molecular analysis of deltamethrin Resistance in the common bed bug (Hemiptera: Cimicidae). J. Med. Entomol..

[10] Reinhardt K., Siva-Jothy M.T. (2007). Biology of the bed bug (Cimicidae). Annu. Rev. Entomol..

[11] Johnson C.G. (1940). Development, hatching and mortality of the eggs of *Cimex lectularius* L. (Hemiptera) in relation to climate, with observations on the effects of reconditioning to temperature. Parasitology.

[12] Gunn W.C. (1933). The Bed Bug (Cimex lectularius): Prevention of House Infestation: A Study of Public Health Purpose.

[13] Johnson C.G. (1941). The ecology of the bed bug, *Cimex lectularius* L., in Britain: Report on research 1935–40. J. Hyg..

[14] Harcourt D.G. (1969). The development and use of life tables in the study of natural insect populations. Ann. Rev. Entomol..

[15] Andrewartha H.G., Birch L.C. (1954). The distribution and Abundance of Animals.

[16] Krebs J.C. (2001). Ecology: The Experimental Analysis of Distribution and Abundance.

[17] Morris R.F., Miller C.A. (1954). Development of life tables for the spruce budworm. Can. J. Zool..

[18] Satpute N.S., Deshmukh S.D., Rao N.G.V., Nimbalkar S.A. (2005). Life tables and the intrinsic rate of increase of *Earias vittella* (Lepidoptera: Noctuidae) reared on different hosts. Int. J. Trop. Insect Sci..

[19] Carey J.R. (1982). Demography and population dynamics of the Mediterranean fruit fly. Ecol. Modell..

[20] Gabre R.M., Adham F.K., Chi H. (2005). Life table of *Chrysomya megacephala* (Fabricius) (Diptera: Calliphoridae). Acta Oecol..

[21] Pilkington L.J., Hoddle M.S. (2007). Use of life tables to quantify reproductive and developmental biology of *Gonatocerus triguttatus* (Hymenoptera: Mymaridae), an egg parasitoid of *Homalodisca vitripennis* (Hemiptera: Cicadellidae). Biol. Control.

[22] Carey J.R. (1983). Practical application of the stable age distributions: Analysis of a tetranychid mite (Acari: Tetranychidae) population outbreak. Environ. Entomol..

[23] Evans F.C., Smith F.E. (1952). The intrinsic rate of natural increase for the human louse, *Pediculus humanus* L. Am. Nat..

[b24-insects-02-00173] 24.Polanco, A.M.; McCoy, T.C.; Miller, D.M. Virginia Tech, Blacksburg, VA, USA, Unpublished work, 2009.

[b25-insects-02-00173] 25.McCoy, T.C.; Miller, D.M. Virginia Tech, Blacksburg, VA, USA, Unpublished work, 2011.

[26] Carey J.R. (1993). Applied Demography for Biologists with Special Emphasis on Insects.

[27] Leslie P.H., Park T. (1949). The intrinsic rate of natural increase of *Tribolium castaneum* Herbst. Ecology.

[28] Birch L.C. (1948). The intrinsic rate of natural increase of an insect population. J. Anim. Ecol..

[29] Pontius J.S., Boyer J.E., Deaton M.L. (1989). Estimation of the stage transition time: Application to entomological studies. Ann. Entomol. Soc. Am..

[30] Roush R.T., Plapp F.W. (1982). Effects of insecticide resistance on biotic potential of the house fly (Diptera: Muscidae). J. Econ. Entomol..

[31] Carriere Y., Deland J.P., Roff D.A., Vincent C. (1994). Life-history costs associated with the evolution of insecticide resistance. Proc. R. Soc. Lond. B.

[32] Groeters F.C., Tabashnik B.E., Finson N., Johnson M.W. (1994). Fitness cost of resistance to *Bacillus thuringiensis* in the diamondback moth (*Plutella xylostella*). Evol..

[33] Roy S., Mukhopadhyay A., Gurusubramanian G. (2010). Fitness traits of insecticide resistant and susceptible strains of tea mosquito bug *Helopeltis theivora* Waterhouse (Heteroptera: Miridae). Entomol. Res..

[34] Georghiou G.P., Taylor C.E. (1986). Factors influencing the evolution of resistance. Pesticide Resistance: Strategies and Tactics for Management.

[35] Banks C.J., Needham P.H. (1970). Comparison of the biology of *Myzus persicae* (Sulzer) resistant and susceptible to Dimethoate. Ann. Appl. Biol..

[36] Hollingworth R.G., Tabashnik B.E., Johnson M.W., Messing R.H., Ullman D.E. (1997). Relationship between susceptibility to insecticides and fecundity across populations of cotton aphid (Homoptera: Aphididae). J. Econ. Entomol..

[37] Noppun V., Miyata T., Saito T. (1984). Decrease in insecticide resistance in the diamondback moth, *Plutella xylostella* L. (Lepidoptera: Yponomeutidae) on release from selection pressure. Appl. Entomol. Zool..

[38] Strong C.A., Koehler P.G., Patterson R.S. (1997). Insecticide resistance decline and slection in laboratory-reared German cockroaches (Dictyoptera: Blattellidae). J. Econ. Entomol..

[39] Geisthardt G. (1937). Ueber die ökologische valenz zweier wanzenarten mit verschiedenen Verbreitungsgebiet. Parasitol. Res..

[40] Taylor F. (1979). Convergence to the stable age distribution in populations of insects. Am. Nat..

[41] Aguilera L., Marquetti M.C., Gutierrez A., Navarro A. (1997). Tablas de vida de *Blattella germanica* (Dictyoptera: Blattellidae) en condiciones de laboratorio y su importancia en el control. Rev. Cubana Med. Trop..

[42] Iversen T., Harding S. (2007). Life table parameters affecting the population development of the woolly beech aphid, *Phyllaphis fagi*. Entomol. Exp. Appl..

